# Competitive Adsorption of Congo red in Single and Binary Systems Using a Low-cost Adsorbent

**DOI:** 10.5696/2156-9614-11.31.210912

**Published:** 2021-08-17

**Authors:** Abdur-Rahim A. Giwa, Isah A. Bello, Mary A. Oladipo, Deborah O. Aderibigbe

**Affiliations:** 1 Department of Pure and Applied Chemistry, Ladoke Akintola University of Technology, Ogbomoso, Nigeria; 2 Department of Chemistry, School of Basic Sciences, Nigeria Maritime University, Warri, Nigeria

**Keywords:** Congo red, equilibrium, isotherm, kinetics, synergistic, thermodynamics

## Abstract

**Background.:**

Sawdust from *Parkia biglobosa* was prepared as an adsorbent for the adsorption of dye from aqueous solution.

**Objectives.:**

The ability of sawdust to adsorb Congo red in a single dye system and binary dye system was examined.

**Methods.:**

Effects of several variables (pH, contact time, initial dye concentration and adsorbent dose) were investigated and optimum conditions were established. The equilibrium data were subjected to kinetic and isotherm models.

**Results.:**

Equilibrium time was observed at 600 mins while the optimum dose was 0.2 g and there was an increase in adsorption at low pH. The equilibrium data fitted the Freundlich isotherm model in both systems (coefficient correlation (R^2^) > 0.9). Harkin-Jura was the worst-fitted (R^2^ < 0.8). The q_m_ in the single system (21.65) was lower than in BDS (27.17; 34.01). The values of 1/n show the heterogeneity on the surface of sawdust which reduced in the BDS. The increase in q_m_ in the binary system showed that the presence of another dye (Reactive black 5) had a synergistic effect on the adsorption of Congo red. The kinetics of the process followed the pseudo-second-order model both in the single system and one of the binary systems. The dynamics of the process showed that the single system was non-spontaneous at low temperatures, endothermic and increased randomness on the surface of the adsorbent, while in the binary system, the process was exothermic and had little affinity for the adsorbent.

**Conclusions.:**

Sawdust from *Parkia biglobosa* is a potential adsorbent for removing Congo red dye from aqueous solution in single and binary systems.

**Competing Interests.:**

The authors declare no competing financial interests.

## Introduction

There is growing concern about widespread contamination of surface and ground water by various organic compounds due to the rapid development of chemical and petrochemical industries over the past several decades. Many industrial wastes contain organics which are difficult or impossible to remove by conventional biological treatment process.^1^

Dyes usually have synthetic origin and complex aromatic molecular structures which make them more stable and more difficult to biodegrade.^2^ There are more than 10 000 commercially available dyes with over 7×10^5^ tons of dyestuff being produced annually across the world.^2^ Dye producers and consumers are interested in the stability and fastness of dyes and consequently are producing dyestuffs which are more difficult to degrade after being used.^3^ Congo red (sodium salt of benzidine diazobis-1-naphthyl-amine-4-sulfonic acid) is a benzidine-based azo dye (an acid dye), a model anionic dye that has a complex chemical structure, high solubility in aqueous solution and difficult to biodegrade or photodegrade because of its stable structure.^4^ The dye is known to be metabolized into benzidine, which is a human carcinogen and mutagen.^5^ Therefore, it is necessary to remove it from bodies of water.

Adsorption has been universally accepted as one of the most effective pollutant removal processes with low cost, ease in handling, low consumption of reagents as well as scope for recovery of value-added components through desorption and regeneration of adsorbent.^6^ Activated carbon is very effective in removal of many impurities occurring in wastewater.

In spite of the high efficiency of commercial activated carbon, because of the high cost and variable performance of carbon regeneration, alternative materials are desirable. This has led many researchers to look for more economic, practical and efficient materials that can perform like commercial activated carbon, which is also the aim of the present study. Adsorption of Congo red has been carried out by using materials such as soil,^7^ rice hull,^8^–^9^ tendu waste,^10^ banana and orange peels,^11^ *Pandanus* leaf^12^ etc. The present study aims to examine removal of Congo red from aqueous solution using adsorbent prepared from sawdust of *Parkia biglobosa* (a common tree found in the location of the study) in aqueous solution. However, in real effluents, it is rare to have only one dye in the solution and since Reactive Black 5 is a common azo dye, it is also paramount to test its effect on the adsorption of Congo red.

## Methods

Sawdust used in the present study was obtained from a local sawmill in Ogbomoso metropolis, southwest Nigeria. Debris and other relatively large foreign materials were hand-picked, then thoroughly washed with distilled water, drained and oven dried. The washed and dried sawdust material was sieved into different particle sizes. It was washed again several times with large quantities of distilled water until the wash water was clear to ensure the removal of any soluble component that may interfere with adsorption processes. It was then oven-dried at 105° C and stored in air-tight containers as sawdust adsorbent.

### Characterization of adsorbent

The surface morphology of the adsorbents was examined on a Hitachi 2300 scanning electron microscope. Samples were coated with gold before being subjected to scanning electron microscope analysis. The Fourier transform infrared (FTIR) spectrum of the sawdust was recorded in the range of 4000 cm^−1^ to 450 cm^−1^ using potassium bromate (KBr) disk as a reference to examine the functional groups on the surface of the adsorbent that could serve as possible binding site. A 5% (wt/wt) suspension of the adsorbent was prepared in double-distilled water and was heated to about 90°C. The slurry was stirred for 20 minutes and allowed to cool. The pH meter was calibrated with pH 4 and pH 7 buffers prior to measurement and the pH measured with the pH-meter probe.

**Abbreviations**
*Q*_*e*_: Quantity adsorbed per unit mass
*R*_*2*_: Correlation coefficient
*SSE*: Sum of square error

### Preparation of adsorbates

Congo red (Color index (C.I.), 22120; lambda max (λ_max_)., 500 nm; molecular weight, 696.66) was supplied by J. T. Baker Inc., Philipsburg, USA and used without further purification. Stock solutions were prepared by dissolving 1000 mg of Congo red in distilled water to make a 1000 mg/L solution. Appropriate working solutions were prepared from the stock solution by accurate dilution with distilled water.

### Batch adsorption studies

Batch adsorption experiments were conducted to study the effects of adsorbent dose, initial concentration of the adsorbate, pH and contact time. The isotherm, kinetics and thermodynamics of the process were also studied. The effect of the mass of sawdust on the adsorption of Congo red was investigated by varying the dose in the range of 0.20–1.0 g in 25 mL of 50 mg/L solution of the dye while keeping other variables (contact time, agitation speed, particle size, temperature) constant in a reaction bottle and were shaken in a horizontal shaker (SM 101 Surgafriend Medicals) at room temperature. This was filtered after equilibrium was established and the residual solution was measured for absorbance at λ_max_., 500 nm using a UV-visible scanning spectrophotometer, Genesys 10. Analysis of the effect of initial concentration was carried out in reaction bottles where the adsorbent dose of 0.2 g was measured into 25 mL of the Congo red solution while varying the concentration of Congo red in the range of 10–100 mg/L at room temperature. This was shaken until equilibrium time was reached and residual solution measured for absorbance. In the binary systems, 5 ppm and 10 ppm of Reactive black was mixed with the Congo red solution.

Experiments were carried out by weighing 0.2 g of the adsorbent in a reaction bottle and 50 mL of a 50 mg/L solution of the dye was introduced at different pH (3–9) using 0.1 M hydrogen chloride (HCl) or 0.1 M sodium hydroxide (NaOH) for the variation while keeping other variables (contact time, agitation speed, particle size, temperature) constant to show the effect of pH on Congo red adsorption. They were shaken in a horizontal shaker (SM 101 Surgafriend Medicals) until equilibrium time was reached and residual solution measured for absorbance. In addition, the effect of contact time on the adsorption process of the dye-sawdust system was studied by shaking 25 mL of 50 mg/L dye solutions with 0.2 g of sawdust adsorbents in several tightly covered reaction flasks at different contact times ranging from 60 to 1080 mins until equilibrium was reached and the residual solution was measured for absorbance. For each adsorption experiment, samples were withdrawn at predetermined time intervals and the dye-adsorbent system was separated by filtration. Then, concentrations of residual solutions were measured by monitoring the absorbance changes at a wavelength of maximum absorbance (λ_max_= 498 nm).

## Results

The results of the characterization of this adsorbent have been reported by the same authors in Aderibigbe *et al*.^13^

### Effect of *Parkia biglobosa* sawdust dose on the adsorption of Congo red

The effect of the mass of sawdust on the adsorption of Congo red was investigated by varying the dose in the range of 0.20–1.0 g in 25 mL of 50 mg/L solution of the dye while keeping other variables (contact time, agitation speed, particle size, temperature) constant and the results are presented in Figure 2. The quantity adsorbed per unit mass (q_e_) ranged between 5.65–0.88 mg/g as the dose increased from 0.2–1.0 g.

**Figure 1:**
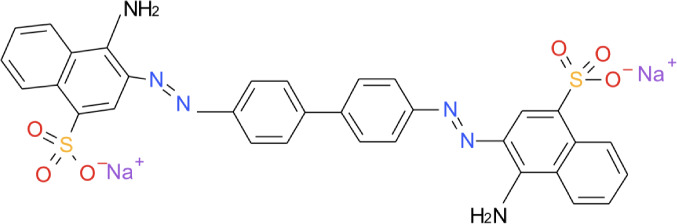
Structure of Congo red

**Figure 2:**
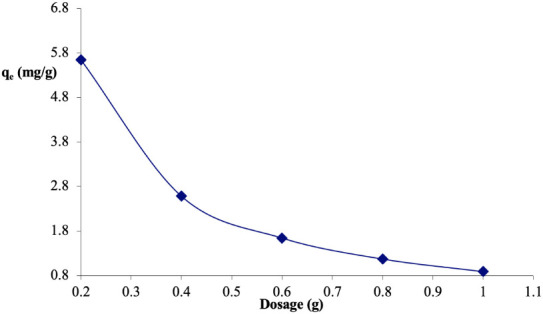
Effect of sawdust of Parkia biglobosa dose on Congo red adsorption

### Effects of initial concentration of Congo red on *Parkia biglobosa* sawdust adsorption capacity

**Figure 3:**
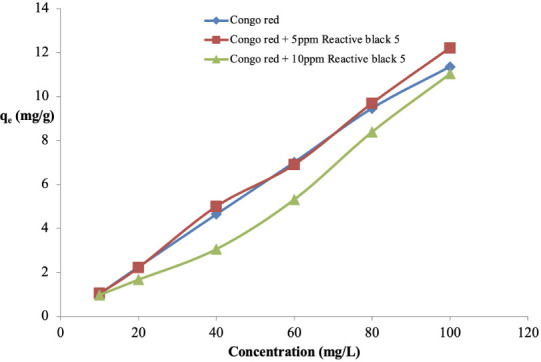
Effect of initial Congo red concentration on its adsorption on sawdust of Parkia biglobosa in single and binary systems

The effect of initial concentration of Congo red on its adsorption onto sawdust was investigated using 25 mL of Congo red at the initial concentrations of 10, 20, 40, 60, 80 and 100 mg/L. Adsorption capacity, q_e_, increased from 1.02 mg/g at a Congo red initial concentration of 10 mg/L to 11.35 mg/g at 100 mg/L initial concentration *(Figure 3).*

### Effect of pH on the adsorption of Congo red

**Figure 4:**
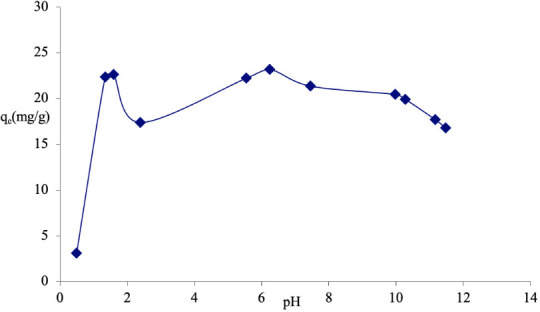
Effect of pH on adsorption of Congo red on sawdust of Parkia biglobosa

Various pH values of Congo red solution (volume 50 mL, concentration 50 mg/L) was prepared to study the adsorption of Congo red on sawdust by adding appropriate amounts of 0.1 M HCl or 0.1 M NaOH. From the result, q_e_ increased from 3.13 to 23.18 mg/g as pH increased from 0.5–6.24 after which it decreased at higher pH *(Figure 4).*

### Effect of contact time on the adsorption of Congo red

**Figure 5:**
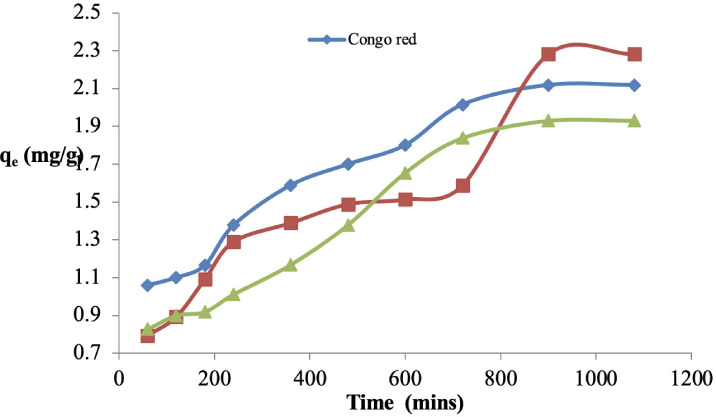
Effect of contact time on the adsorption of Congo red on sawdust of Parkia biglobosa in single and binary systems

The effect of contact time between sawdust and the Congo red solution was studied by varying the time from 60–1080 mins. The quantity adsorbed increased from 1.16–2.75 mg/g as the contact time increased from 60–1080 mins. There was not much difference between the single system and the two binary systems except that there was slight reduction in q_e_ in the binary systems *(Figure 5).*

**Figure 6:**
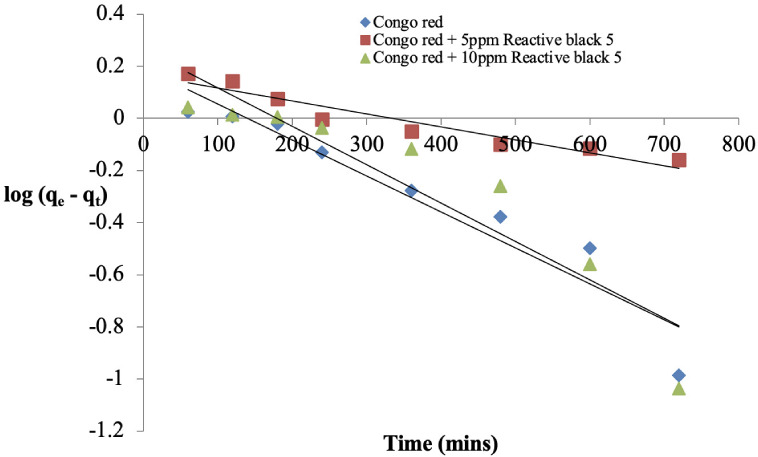
Pseudo-first order kinetic plot for the adsorption of Congo red onto sawdust of Parkia biglobosa in single and binary systems

**Figure 7:**
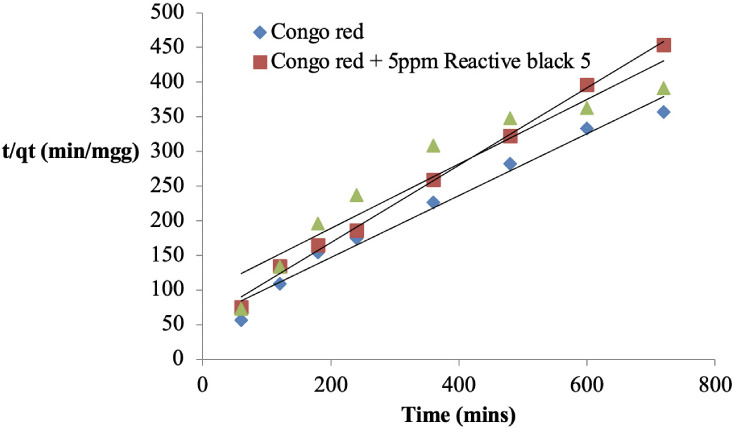
Pseudo-second order kinetic plot for the adsorption of Congo red onto sawdust of Parkia biglobosa in single and binary systems

**Figure 8:**
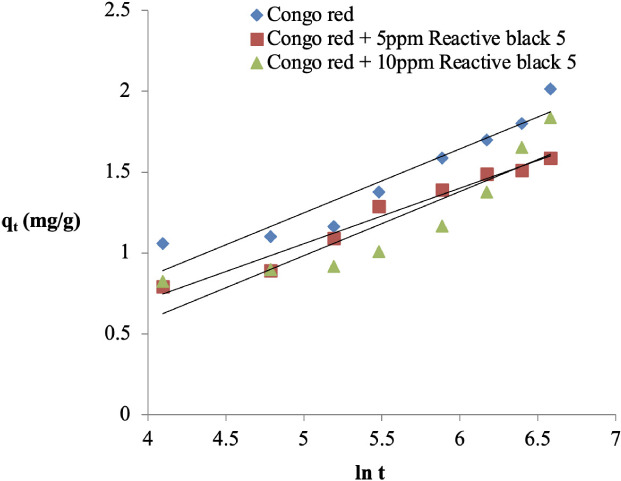
Elovich kinetic plot for the adsorption of Congo red onto sawdust of Parkia biglobosa in single and binary systems

**Figure 9:**
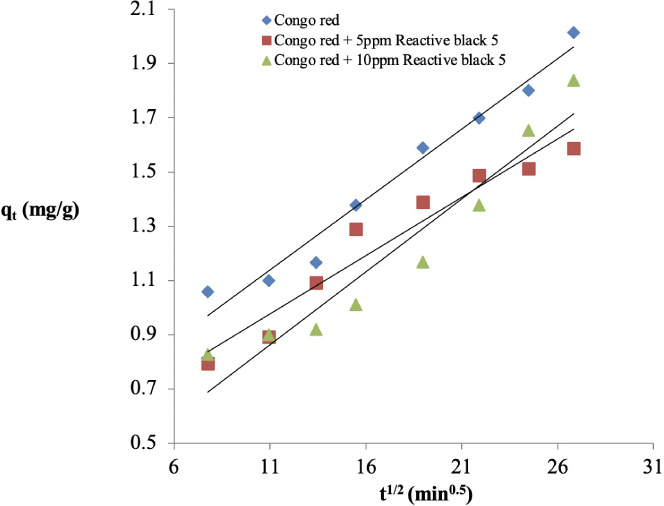
Intra-particle diffusion plot for the adsorption of Congo red onto sawdust of Parkia biglobosa in single and binary systems

### Kinetics of Congo red adsorption onto *Parkia biglobosa* sawdust

The kinetic data was fitted into four models and they are presented in Figure 5 while the kinetic parameters are presented in Table 1. The three most widely used kinetic models; Pseudo-first-order,^14^ Pseudo-second-order^15^ and the Elovich^16^ model were used to test the kinetic data of the adsorption of Congo red onto sawdust, while Weber-Morris^17^ was used to determine if diffusion is the rate determining step of the process. The coefficient correlation (R^2^) of the four models used was higher than 0.9 but in the binary systems it was between 0.8 and 0.9 *(Table 1).* In addition, the experimental quantity adsorbed at equilibrium (q_e(expt)_) in the single system was 2.12 mg/g, while that of the binary systems were 2.28 and 1.93 mg/g, respectively. The sum of square error (SSE) was less than 0.5 for the five models in the two systems.

**Table 1:** Comparison of Rate Constants and other Parameters across Various Kinetic Models for Congo Red Adsorption in Single and Binary Systems

**Models/variables**	**Congo red only**	**Congo red** + **5ppm Reactive black 5**	**Congo red+10ppm Reactive black 5**
q_e(expt)(mg/g)_	2.12	2.28	1.93

Pseudo-first-order			
R^2^	0.913	0.918	0.856
K_1_ (min^−1^)	2.30 × 10^−3^	0.000	2.30 × 10^−3^
q_e_ (calc) (mg/g)	1.56	1.47	1.84
SSE (%)	0.20	0.29	0.033
Pseudo-second-order			
R^2^	0.977	0.996	0.907
K_2_(L/mg.min)	3.45 × 10^−3^	5.53 × 10^−3^	2.25 × 10^−3^
q_e_ (cal)(mg/g)	2.24	1.79	2.15
h (mg/g.min)	0.017	0.018	0.0104
SSE (%)	0.042	0.173	0.077
Elovich model			
R^2^	0.906	0.970	0.811
A	0.0627	0.0503	0.0318
B	2.53	2.91	2.53
SSE (%)	0.058	0.19	0.029
Weber-Morris			
R^2^	0.971	0.941	0.924
K_id_ (mgg^−1^min^0.5^)	0.052	0.053	0.053
X_i_	0.566	0.504	0.272
SSE (%)	0.031	0.082	0.019

Abbreviations: R^2^, correlation coefficient; K_1_. rate constants for pseudo-first order; q_e_, quantity adsorbed per unit mass; SSE, sum of square error; h, initial adsorption rate as t approaches 0; α, initial adsorption rate; β, desorption constant; K_d_ intraparticle diffusion rate constant; X_i_, boundary layer thickness

### Isotherms of Congo red adsorption onto *Parkia biglobosa* sawdust

Langmuir,^18^ Freundlich,^19^ Temkin,^20^ Dubinin-Radushkevich^21^ and Harkin-Jura^22^ isotherm models were used to describe the isotherm for the adsorption of Congo red onto sawdust. The equilibrium data were fitted into the five models and the isotherm variables are presented in Table 2. The R^2^ of the five models for a single system ranged from 0.788 to 0.984 while in the binary systems it ranged from 0.672 to 0.999. The maximum monolayer coverage (q_m_) was 21.65 mg/g in a single system and 27.17 and 34.01 mg/g in the two binary systems, respectively *(Table 2).*

**Table 2:** Isotherm Model Variables for Adsorption of Congo Red onto Sawdust of Parkia biglobosa

**Model/variables**	**Congo red only**	**Congo red + 5 ppm Reactive black 5**	**Congo red +10 ppm Reactive black 5**
Langmuir			
R^2^	0.981	0.867	0.961
q_m_(mg/g)	21.65	27.17	34.01
K_a_(L/mg)	0.0328	0.0264	1.000
Freundlich			
R^2^	0.984	0.979	0.999
1/n(L/g)	0.727	0.830	0.867
K_f_(mg/g)	0.943	0.787	0.491
Temkin			
R^2^	0.982	0.948	0.920
B (kJ/mol)	4.101	3.739	3.503
K_T_ (L/mg)	0.425	1.609	0.417
Dubinin-Radushkevich			
R^2^	0.882	0.847	0.742
β (mol^2^/J^2^)	4×10^−6^	2×10^−6^	2×10^−6^
X_m_(mg/g)	83.07	77.68	97.29
Harkin — Jura			
R^2^	0.788	0.672	0.735
A	5.371	1.644	1.342
B	1.411	1.253	1.362

Abbreviations: q_m_, monolayer adsorption coverage, K_a_, equilibrium constant, n, sorption intensity constant, K_f_, sorption capacity constant, B, heat of adsorption, K_T_, equilibrium binding constant, β, activity coefficient related to mean sorption energy, X_m_, maximum adsorption capacity

### Thermodynamics for the adsorption of Congo red onto sawdust

A plot of ln K_C_ versus 1/T for the adsorption of Congo red onto sawdust for all the systems gives a straight line and the thermodynamic parameters (Gibbs free energy change (ΔG), enthalpy change (ΔH) and entropy change (ΔS)) are shown in Table 3. The Gibbs free energy change (ΔG) ranged from +6.04 to +5.49 kJ/mol as the temperature increased from 303 to 323K while the enthalpy change (ΔH) was +14.41 kJ/mol in the single system. Meanwhile in the two binary systems, ΔG ranged between 5.57 and 6.20 kJ/mol *(Table 3).*

**Table 3:** Thermodynamic Variables for the Adsorption of Congo red in Single and Binary Systems on Sawdust of Parkia biglobosa

**System/ Parameters**	**303K**	**313K**	**323K**
Congo red			
ΔG (kJ/mol)	+6.04	+5.69	+5.49
ΔH (kJ/mol)	+14.41		
ΔS (J/mol.K)	+0.03		
Congo red + 5ppm			
Reactive black 5			
ΔG (kJ/mol)	+6.16	+6.18	+6.20
ΔH (kJ/mol)	−5.45		
ΔS (J/mol.K)	−0.0023		
Congo red + 10ppm			
Reactive black 5			
ΔG (kJ/mol)	+6.16	+5.57	+5.58
ΔH (kJ/mol)	+15.06		
ΔS (J/mol.K)	+0.029		

Abbreviations: ΔG, Gibbs free energy change; ΔH, enthalpy change; ΔS, entropy change

## Discussion

Figure 2 shows the dependence of the adsorption capacity of sawdust with its mass. It was observed that the quantity of Congo red removed per unit mass of sawdust adsorbent decreased with increasing mass of sawdust. For instance, adsorption capacity decreased from 5.69 mg/g to 0.89 mg/g with an increase in sawdust dosage from 0.20 g to 1.00 g. This trend may be attributed to the splitting effect of the concentration gradient between adsorbate and adsorbent with increasing dosage of sawdust; leading to a decreasing amount of Congo red adsorbed per unit mass of the adsorbent.^4^,^23^ This was also observed by other researchers.^3^,^4^,^13^,^24^

### Effects of initial concentration of Congo red on sawdust adsorption capacity

The results shown in Figure 3 indicate that there is an increase in q_e_ as concentrations were increased. This could be explained by the fact that as concentrations increased, the mass gradient pressure between the dye and the adsorbent increased, which served as a driving force for the transfer of dye molecules into the surface of the particles of the adsorbent. There was no significant difference in the two binary systems. This was also observed in the adsorption of Congo red on activated carbon prepared from Pandanus leaves^12^ and chemically modified papaya seeds.^25^

### Effect of pH on the adsorption of Congo red

There was an increase in adsorption of Congo red in the lower pH (acidic region) region which may be attributed to the protonation which resulted from an increase in blockage of the sawdust surface by water molecules associated with a negative charge and the positive charge on Congo red occupying the active adsorption sites (*Figure 4*). On the other hand, the adsorption capacity decreased in the alkaline medium which may be as a result of repulsion between the positive ions remaining on the adsorption sites of sawdust and the positive charge of Congo red. This is in line with what was observed when Congo red was adsorbed using adsorbent prepared from banana and orange peels.^10^

### Effect of contact time on the adsorption of Congo red

The amount of Congo red adsorbed per unit mass (q_t_) of sawdust increased as contact time increased at a rapid rate at the beginning until equilibrium was attained and a slow increase afterwards *(Figure 5).* For example, the amount of Congo red removed at 60 minutes contact jumped from 1.06 mg/g to 1.38, 1.59, 1.70 and 1.80 mg/g, at 240, 360, 480 and 600 minutes, respectively, until a 95% removal percentage was attained at about 720 minutes *(Figure 5).* There was not much difference between the single system and the two binary systems except that there was slight reduction in q_e_ in the binary systems, indicating that the presence of another dye reduced the amount adsorbed but the percentage removal is still the same. This was also observed in a previous study.^25^

### Kinetics of Congo red adsorption onto sawdust

In the single system, whereby Congo red only is present in the medium, the R^2^ value of the pseudo-second-order model was the highest (0.987) compared to that of the pseudo-first-order and Elovich model (0.599 and 0.935, respectively). The low SSE value (0.091) is further support that the pseudo-second-order model best explained the process *(Table 1).* In the binary system, in which Congo red was adsorbed in the presence of 5 ppm Reactive black 5, the high R^2^ value of the pseudo-first-order model (0.918) showed that the process followed this model, while those of the pseudo-second-order model and the Elovich model were lower (0.896 and 0.843, respectively). The low value of SSE also supports this *(Table 1).*

However, in the binary system, in which Congo red was adsorbed in the presence of 10 ppm Reactive black 5, the R^2^ value of the pseudo-second-order model was the highest followed by the Elovich model and the pseudo-first-order model *(Table 1).* The low SSE value also indicates that the process followed the pseudo-second-order model *(Table 1).* In all the systems, diffusion is not the only rate limiting step in the process. A similar observation was reported in the literature.^4^

### Isotherms of Congo red adsorption onto sawdust

In the single system, of all the five isotherm models, the Freundlich model gave the best fit, with a higher R^2^ (0.984) than the others. Temkin and Langmuir models can also be used to explain the adsorption with an R^2^ of 0.982 and 0.981, respectively, while Dubinin-Radushkevich and Harkin-Jura were least able to describe the process with R^2^ of 0.882 and 0.788, respectively. In the binary system, where adsorption of Congo red took place in the presence of 5 ppm Reactive black, the order of fitness and R^2^ was as follows: Freundlich (0.979) > Temkin (0.948) > Langmuir (0.867) > Dubinin-Radushkevich (0.847) > Harkin-Jura (0.672).

In addition, in the binary system, where adsorption of Congo red was observed in the presence of 10 ppm Reactive black, the Freundlich model could best describe the process, with a high R^2^ of 0.999. The Langmuir and Temkin model can also explain the process with an R^2^ of 0.961 and 0.920, respectively, while the process could least be described by the Dubinin-Radushkevich and Harkin-Jura models as seen from their low R^2^ of 0.742 and 0.735, respectively. The K_R_ value for all the systems was between 0 and 1, which showed that the process is favorable. Overall, the adsorption of Congo red onto sawdust could be well explained by the Freundlich isotherm model. A similar report was given in the literature.^4^,^26^

### Thermodynamics for the adsorption of Congo red onto sawdust

In the single system, ΔG was positive, indicating the non-feasibility of the process at lower temperatures, and ΔH was positive, indicating an endothermic process in which heat can speed up the rate of reaction and ΔS was positive showing good affinity of the adsorbate-adsorbent system. In addition, in the binary system of Congo red and 10 ppm Reactive black 5, ΔG, ΔH, and ΔS were positive, showing the same trend as that of the single system. In the binary system of Congo red and 5 ppm Reactive black 5, ΔG was positive and ΔH was negative, showing the process is exothermic, and ΔS was negative, indicating that there is little affinity between the adsorbate and the adsorbent, and there was a decrease in the randomness of the adsorption on the surface of sawdust. This is similar to what has been reported in the literature.^13^

## Conclusions

The present study investigated the potential of sawdust from the *Parkia biglobosa* tree in removing Congo red from aqueous solution, especially in a competitive environment (mixture with 5 ppm and 10 ppm Reactive black 5). It was observed that at 600 mins the process reached the optimum level after which equilibrium was established. The optimum dose was 0.2 g and there was increase in adsorption at low pH. Adsorption increased when initial dye concentration increased. The data fitted the isotherm model in the order of Freundlich > Langmuir > Temkin > Dubinin-Radushkevich > Harkin-Jura in the single system, while in the binary systems, it followed the Freundlich model closely (R^2^ = 0.999) and the Harkin-Jura model was the worst-fitted (R^2^ = 0.687). The K_R_ values showed that the process was favorable. The kinetics of the process followed the pseudo-second-order model both in the single system and one of the binary systems. The other binary system had pseudo-first-order as the best fit. Both systems showed that the process was dependent on film diffusion and not only pore diffusion with a high R^2^ and low SSE for the Weber-Morris model and the boundary layer X_i_ increased in the binary systems. The dynamics of the process showed that the single system was non-spontaneous at low temperatures, endothermic and increased randomness on the surface of the adsorbent, while in the binary systems, the process was exothermic and had little affinity for the adsorbent.
